# Nutritional Psychiatry: A Novel Approach to the Treatment of Mental Health Disorders

**DOI:** 10.62641/aep.v53i2.1920

**Published:** 2025-03-05

**Authors:** Alejandro Borrego-Ruiz, Juan J. Borrego

**Affiliations:** ^1^Departamento de Psicología Social y de las Organizaciones, Universidad Nacional de Educación a Distancia (UNED), 28040 Madrid, Spain; ^2^Departamento de Microbiología, Universidad de Málaga, 29071 Málaga, Spain

Mental disorders can be understood as psychological patterns marked by clinically significant distress or impairment, reflecting underlying psychobiological dysfunctions rather than being merely expected responses to common stressors or sociocultural factors, while also emphasizing diagnostic validity, clinical utility, and differentiation from closely related conditions, as proposed in the Diagnostic and Statistical Manual of Mental Disorders, Fifth Edition (DSM-5) [1]. An estimated 970 million individuals worldwide live with a mental health disorder, with symptoms frequently emerging in childhood, adolescence, or young adulthood, and often persisting throughout life, impairing functioning and quality of life [2]. More specifically, in 2019, 11.63% of individuals aged 5 to 24 globally were affected, with anxiety-related conditions being the most prevalent [3]. In this regard, prevalence increased with age, especially in mood-related conditions, and exhibited pronounced sex-specific patterns [3]. Nevertheless, the distinction between individual and societal impact, the need for clearer indicators of dysfunction, and the inclusion of valid diagnostic criteria in the definition of mental disorders, along with further clarification of the type and extent of data required to support these aspects, should also be considered [1].

The clinical strategies typically applied for the treatment of mental disorders include pharmacotherapies and psychotherapies, which have shown modest overall benefits but often with small effect sizes and significant heterogeneity across conditions [4]. The drug discovery process for addressing mental health disorders also faces significant challenges, including high failure rates, long development times, and misalignment with current diagnostic systems, further highlighting the difficulties in developing more effective treatments [2]. Moreover, the pharmacological agents employed and their administration strategies within psychiatric care settings can present distinct safety concerns. For instance, psychotropic medications, including benzodiazepines, antidepressants, and antipsychotics, are commonly utilized in hospitalized psychiatric patients and are associated with adverse physiological health effects, such as dyslipidemia, altered glucose metabolism, extrapyramidal symptoms, metabolic syndrome, weight gain, type 2 diabetes, sexual dysfunction, as well as respiratory and cardiovascular diseases [5]. In addition, the use of high-dose antipsychotics, along with interactions among drugs or between drugs and preexisting conditions, may increase the risk of medication-related complications [5]. Therefore, all these factors underscore certain limitations of current approaches and suggest the potential need for a paradigm shift in research and treatment strategies.

From a mechanistic point of view, a possible direct antioxidant activity, anti-inflammatory effect, and/or functional modulation may strengthen the theoretical basis for dietary influences on mental health [6], thereby supporting the development of a new discipline called nutritional psychiatry [7]. Thus, nutritional psychiatry encompasses the study of dietary and nutrient-based interventions for the prevention or treatment of mental disorders. The concept of nutraceuticals refers to non-toxic dietary extracts or supplements with scientifically validated benefits for promoting health and aiding in disease management [8]. Nutraceuticals derive their effects from essential nutrients, such as fatty acids, carbohydrates, proteins, vitamins, and minerals, as well as from non-essential bioactive components, such as folates, phenols, polyamines, flavonoids, anthocyanins, ellagitannins, and carotenoids. These components modulate several cellular processes (e.g., antioxidant processes, gene expression, cell proliferation, mitochondrial integrity, immune enhancement) and provide protection against diverse health-related conditions, including cancer, obesity, type 2 diabetes, microbial infections, as well as cardiovascular, gastrointestinal, and neurodegenerative diseases [9]. There is increasing evidence indicating that high intakes of nutraceuticals such as fiber, phytochemicals, and omega-3 fatty acids are associated with overall mental health and with the prevention of neurodevelopmental disorders [10]. Moreover, recent advances in understanding their molecular mechanisms reveal that deficiencies in several nutrients, such as amino acids (e.g., tryptophan, tyrosine, phenylalanine, and methionine), vitamins (e.g., B_6_, B_12_, and folate), minerals (e.g., zinc, magnesium, copper, iron, selenium, and lithium), fibers (i.e., through the secretion of short-chain fatty acids by the gut microbiome), and omega-3 fatty acids (e.g., linolenic, eicosapentaenoic, and docosahexaenoic acids), have been linked to the onset of specific mental disorders [10]. Nevertheless, while the aforementioned associations are promising, deeper insights into the underlying processes and the optimization of clinical evaluations are required in order to obtain clear causal relationships and more definitive conclusions.

Brain development and functionality rely on the availability of essential nutrients, making it reasonable to infer that dietary patterns can influence brain-related activity. This establishes diet as a modifiable factor for addressing cognitive performance, mood, and mental health issues [11]. Furthermore, neurotransmitters, neuropeptides, endogenous gut hormones, and the gut microbiome are all directly impacted by the specific nutritional composition of the diet. Within this framework, the gut microbiome plays a pivotal role in maintaining the functional and structural integrity of the gastrointestinal system. Certain dietary components can alter the gut microbiome, leading to the compromise of the gut barrier against toxins, microbial metabolites, and pathogens, as well as to changes in nutrient absorption, the onset of chronic inflammation, and subsequent activation of neural pathways that directly influence central nervous system functionality via the gut-brain axis [11]. However, the understanding of the role of the dietary components regarding mental health requires addressing several questions, such as how diet affects: (i) metabolic processes in the gut microbiome; (ii) gut-brain signaling; (iii) levels of metabolites in the blood and target organs; (iv) cellular and neural network responses; (v) genetic background effects on the impact of dietary patterns on mental health; and (vi) gene expression and downstream effects [12].

In recent years, the field of nutritional psychiatry has been increasingly shaping clinical practice, emphasizing the need to employ scientifically rigorous methods to evaluate efficacy and define appropriate therapeutic applications, given the extensive prevalence of dietary supplements and nutraceuticals among individuals with and without mental health conditions. Simplistic research focusing on isolated nutrients provides limited value to the field, and recent data suggest that studies on broad-spectrum, “shotgun” nutraceutical formulations are similarly unproductive. Therefore, the next pivotal step in the field could involve designing psychiatric intervention studies focused on dietary modifications and nutraceutical applications that align more closely with a personalized medicine framework, which could be carried out in conjunction with established treatments such as pharmacotherapy or psychotherapy, positioning nutritional approaches as novel complementary or supportive strategies. This approach should incorporate biomarkers such as levels of inflammatory cytokines, nutrient deficiencies, genomic profiles, analysis of the microbiome composition, insights on dietary patterns and individual nutrient requirements, as well as indicators reflecting the interaction between traditional therapies and dietary factors [13]. Furthermore, the combined supplementation of psychobiotics (i.e., probiotics or microbial derivatives that confer mental health benefits to the host) and nutraceuticals might represent a synergistic approach to treating certain psychiatric conditions [14]. However, future research must address the gap between epidemiological findings and clinical evidence regarding the management of mental disorders through diet-related factors, which can be achieved by investigating mechanistic pathways involving the gut microbiome and its interplay with the central nervous system [15].

In conclusion, the progress in nutritional psychiatry shows significant potential, but it is essential to recognize that this field remains in its early stages, requiring robust, multidisciplinary investigations to establish its role as a consistent and integrative approach for the treatment of mental health disorders. In this regard, translating microbiota-related findings into clinical practice poses substantial challenges, including the complexity of individual microbiomes and the difficulty in determining causal links between dietary interventions and clinical outcomes. Furthermore, while dietary interventions can be a valuable supportive strategy, they are not curative for severe mental illnesses and should always be considered as part of a comprehensive treatment plan that includes other approaches that have been more extensively validated.

## Availability of Data and Materials

Not applicable.

## References

[1] Stein DJ, Palk AC, Kendler KS (2021). What is a mental disorder? An exemplar-focused approach. *Psychological Medicine*.

[2] Scangos KW, State MW, Miller AH, Baker JT, Williams LM (2023). New and emerging approaches to treat psychiatric disorders. *Nature Medicine*.

[3] Wu Y, Wang L, Tao M, Cao H, Yuan H, Ye M (2023). Changing trends in the global burden of mental disorders from 1990 to 2019 and predicted levels in 25 years. *Epidemiology and Psychiatric Sciences*.

[4] Leichsenring F, Steinert C, Rabung S, Ioannidis JPA (2022). The efficacy of psychotherapies and pharmacotherapies for mental disorders in adults: an umbrella review and meta-analytic evaluation of recent meta-analyses. *World Psychiatry: Official Journal of the World Psychiatric Association (WPA)*.

[5] Alshehri GH, Keers RN, Ashcroft DM (2017). Frequency and Nature of Medication Errors and Adverse Drug Events in Mental Health Hospitals: a Systematic Review. *Drug Safety*.

[6] Godos J, Currenti W, Angelino D, Mena P, Castellano S, Caraci F (2020). Diet and Mental Health: Review of the Recent Updates on Molecular Mechanisms. *Antioxidants (Basel, Switzerland)*.

[7] Logan AC, Jacka FN (2014). Nutritional psychiatry research: an emerging discipline and its intersection with global urbanization, environmental challenges and the evolutionary mismatch. *Journal of Physiological Anthropology*.

[8] Aronson JK (2017). Defining ‘nutraceuticals’: neither nutritious nor pharmaceutical. *British Journal of Clinical Pharmacology*.

[9] Puri V, Nagpal M, Singh I, Singh M, Dhingra GA, Huanbutta K (2022). A Comprehensive Review on Nutraceuticals: Therapy Support and Formulation Challenges. *Nutrients*.

[10] Bozzatello P, Novelli R, Montemagni C, Rocca P, Bellino S (2024). Nutraceuticals in Psychiatric Disorders: A Systematic Review. *International Journal of Molecular Sciences*.

[11] Borrego-Ruiz A, Borrego JJ (2025). Human gut microbiome, diet, and mental disorders. *International Microbiology: the Official Journal of the Spanish Society for Microbiology*.

[12] Adan RAH, van der Beek EM, Buitelaar JK, Cryan JF, Hebebrand J, Higgs S (2019). Nutritional psychiatry: Towards improving mental health by what you eat. *European Neuropsychopharmacology: the Journal of the European College of Neuropsychopharmacology*.

[13] Sarris J (2019). Nutritional Psychiatry: From Concept to the Clinic. *Drugs*.

[14] Borrego-Ruiz A, Borrego JJ (2024). Psychobiotics: A new perspective on the treatment of stress, anxiety, and depression. *Anxiety and Stress*.

[15] Grosso G (2021). Nutritional Psychiatry: How Diet Affects Brain through Gut Microbiota. *Nutrients*.

